# Manual Strangulation

**Published:** 1835

**Authors:** S. D. Gross

**Affiliations:** Professor of General and Pathological Anatomy, Physiology, and Medical Jurisprudence in Cincinnati College; Cincinnati


### II.—Manual Strangulation.

*Observations on Manual Strangulation, illustrated by Cases and Experiments.* By S. D. Gross, M. D., Professor of General and Pathological Anatomy, Physiology, and Medical Jurisprudence in Cincinnati College.

The subject of manual strangulation, although one of deep interest in a medico-legal point of view, has not hitherto received that attention from the profession which its great importance demands. Indeed, in many of our systematic works on medical jurisprudence, it is either but very imperfectly discussed, or totally neglected, most authors evidently considering it as a topic of comparatively little consequence. The reason of this is not very obvious, unless we seek for it in the fact that no writer, either ancient or modern, has deemed the subject of sufficient dignity and importance to make it the object of special investigation, and thereby raise it to that rank which it ought to hold in every treatise on forensic medicine.

Manual strangulation may be defined to be a species of murder, in which, as its name implies, death is produced, either directly or indirectly, by the pressure of the hand, applied upon the larynx or trachea, so as to interrupt the respiratory process. It is probably a more frequent but less violent mode of homicide than hanging, and hence the symptoms are often extremely obscure, or so blended with those characterizing strangulation by other causes, that it is by no means easy always to discriminate between them.

In every species of strangling, whether it be the result of hanging or of manual effort, the general appearances exhibited on dissection are very nearly alike, the only differences consisting in the external lesions which we may expect to meet at the part of the body on which the violence has been inflicted. In most cases there is a distortion of the countenance, attended with lividity, and more or less swelling; the eyes are suffused with blood, and propelled, as it were, from their sockets; the lids are half open, and of a bluish color; the tongue is tumid, livid, and protruded; the lips are of a dark purple hue; and the mouth and nose contain more or less sanguinolent froth. In hanging, the cartilages of the larynx are also sometimes fractured; and the vertebræ of the neck not unfrequently dislocated. Luxation is particularly apt to take place in heavy persons, or in those who fall from a height upon the end of the rope, or where attempts are made to hasten death by augmenting the weight of the body. These accidents, it is obvious, will not be so likely to occur in manual strangulation, since the amount of pressure necessary to occlude the windpipe, need neither be so great nor so violent, in order to induce death, as under the circumstances just mentioned. The likelihood of an occurrence of this kind will be still less probable if, at the time of the murder, the neck be resting upon a level surface, in which case it would be almost impossible, however great the force applied to the throat, to break or luxate any of the cervical vertabræ.

In both sorts of death the violence is ordinarily so great as to give rise to the expulsion of the urine, fœces, and semen. In persons that have been hung, the neck presents a livid, discolored aspect, and the impression of the cord is generally very distinct, extending horizontally round the cervical region. In persons, on the contrary, that have been strangled by the hand, we usually find marks of violence in front of the throat, caused by the fingers or some other body. In either case the appearances here alluded to are in general sufficiently characteristic to enable us to distinguish, without difficulty, the sort of death by which the individual has been destroyed. In addition to these symptoms there may be wounds or abrasions on different parts of the body; and, in most instances, the fingers are bent, and the hand nearly closed.

On examining the interior parts of the body, it will generally be found that they are inordinately loaded with blood, especially the lungs and the heart; the large vessels of the neck are likewise greatly distended; the mucous membrane of the mouth, fauces, and respiratory organs, is deeply injected; and the air passages usually contain more or less bloody froth. These signs, however, as well as some of those observable on the exterior of the body, are extremely variable, and as it is of importance to know what amount of confidence should be placed upon each, I shall again advert to them in a subsequent part of this paper.

In regard to the immediate causes of this species of death, writers are by no means agreed. One of the most generally received opinions is, that which supposes it to be effected by a kind of apoplexy, produced by pressure on the large veins of the neck. The blood being thus prevented from returning to the heart, must necessarily give rise to a certain degree of cerebral compression, followed speedily by drowsiness, and deep coma. Owing to this fact, persons who are hung or strangled seldom suffer much pain, and if the compression of the windpipe and the vessels of the neck be not too long continued, or blood extravasated into the brain, recovery in such cases would neither be impossible nor probably of unfrequent occurrence. The celebrated Morgagni mentions the case of a person that survived hanging, who remembered nothing of what transpired, excepting a few flashes of light that passed before his eyes at the moment he was swung off from the cart: he experienced no pain, and soon sunk into a most profound sleep. Similar instances are narrated by Wepfer, Fodere, and other writers.

A second, and by far more rational opinion is that which attributes the cause of death to the occlusion of the windpipe, by which the air is prevented from reaching the lungs. In consequence of this the blood is deprived of the influence of the atmosphere, and being thus rendered incapable of stimulating the brain and heart, these organs speedily cease to execute their functions.

Pressure on the pneumo-gastric and sympathetic nerves, has also been supposed by some to be a prominent effect in producing death in strangulation, by interrupting the nervous influence; but as these structures cannot be compressed without at the same time arresting the circulation in the large vessels of the neck, it is evident that it cannot be entitled to the importance that has been attributed to it.

A fourth cause, and the last to which I shall here allude, is a luxation or fracture of one or more of the upper cervical vertebræ, arresting respiration by destroying the functions of the phrenic nerves. This accident, however, as already stated, is not likely to take place in manual strangulation, though in hanging it is of frequent occurrence, and of course always fatal.

Having thus presented a rapid sketch of the general signs of the several species of strangulation, with a few of the more plausible opinions, as to the proximate causes of this kind of death, I shall proceed, in the next place, to relate the case of Mrs. Getter, who was killed by manual strangulation, in 1833.

Mrs. Getter was murdered on the night of the 27th of February, in an open field, about two miles from Easton, Pennsylvania, her husband being the perpetrator of the deed. Her body was found the next morning, with the head lying in a somewhat dependent position; and the dissection was made on the same day, about two o'clock in the afternoon, at a house in the neighborhood, whither she had been removed. She was a woman of middle stature, with a short, thick neck, and a broad, expanded chest, her whole appearance being remarkably strong and robust. At the time of my examination, the body was stiff but not frozen, although the weather had been quite cold the previous night. The countenance exhibited a full, bloated aspect; the lips were tumid, and of a dark bluish tint; the tongue was slightly livid, but did not project between the teeth; the mouth was filled with froth, and the vessels of its lining membrane greatly distended; the jaws were nearly closed; and the nostrils plugged up with solid ice, caused no doubt by the congelation of the frothy discharges that took place soon after death; the eyes were extremely prominent, and the lids half open, and turgid, their connecting membrane — the conjunctiva — being unusually vascular. The ears and temples, especially the left, were of a dark color, the subcutaneous veins of those parts being evidently engorged with blood. The hair was dishevelled, and several of the teeth of the comb, which was broken, were entangled in it. The throat exhibited marks of violence, on the right side was an indentation, deeper in the middle than at the extremities, which had the appearance as if it had been made by a thumb or finger nail; it extended into the true skin, and was about half an inch long, by the twelfth of an inch in width. An abrasion also existed at the upper part of the larynx, passing from near the middle line of the neck over towards the left side, its breadth being about an inch and a half, and its vertical extent about one inch. On removing her clothes, I likewise discovered an abrasion on the left shoulder, but it was so slight as not to indicate any serious injury. Such were the appearances, and such the marks that were found on the exterior of the body; I shall now proceed to describe those that presented themselves on dissection.

On laying open the neck the veins bled profusely at every incision, rendering it necessary for me to make frequent use of the sponge; the jugulars, indeed, both external and internal, were distended to their very utmost. The carotid arteries of both sides were empty. Beneath the muscles on the left side of the neck, towards the upper part of the larynx, was a slight extravasation of blood, corresponding to the external marks already alluded to, as situated at that portion of the cervical region. The windpipe was filled with frothy matter, and its lining membrane throughout deeply injected: there was no fracture of the cartilages of the larynx, nor any appearance of injury in those of the trachea.

The diaphragm was arched; and the lungs were throughout of a deep black, their vessels bleeding freely on being cut into: the blood which flowed from them was of a dark venous color, mixed with froth. They covered the sides of the heart, and were, in every other respect, perfectly natural.

The cavities of the heart, both right and left, were filled with dark blood, the auricles being almost black. The coronary vessels were greatly injected, and altogether the organ presented the appearance of a beautiful vascular preparation. A small quantity of watery fluid was contained in the pericardium, the vessels of which were likewise somewhat engorged.

The abdominal viscera were, in every respect, natural, as well as healthy, none of them presenting the slightest structural derangement. The uterus, the vessels of which were elegantly injected, contained a fœtus of about seven months. The bladder was empty, and contracted.

It will be perceived from the details of the above case, that the brain of the deceased was not examined; this omission I had afterwards reason much to regret, not indeed on account of any doubt raised in my own mind concerning the true cause of her death, but on account of the fact that we should never neglect, in investigations of this kind, the most trivial circumstances that may be calculated to throw light on the subject, or prevent the counsel of the prisoner from taking undue advantage of what, to him, might appear great ignorance on the part of the medical witness. Anxious to compensate, in some degree, for this omission, I was induced, shortly after having examined the body of Mrs. Getter, to institute a series of experiments, a few only of which it will be necessary here to relate.

*Experiment* 1.—Strangled a small dog, by applying the hands firmly over the superior part of the larynx. A few moments after the pressure was commenced, the animal discharged his urine and fœces; the tongue assumed a dark bluish color, but did not protrude through the mouth; the eyes became dilated; and in ten minutes the dog was completely dead, the pulsation having ceased both in the arteries and in the heart.

*Dissection six hours after death.*—Body stiff—pupils widely dilated—tongue of a dark livid color—small quantity of froth in the fauces, but none at the nose or mouth. *Neck.*—Veins greatly distended, being literally filled with black fluid blood—both carotids empty—mucous membrane of the larynx, trachea, and bronchial tubes almost white, there being here and there merely a little capillary injection—no froth in these passages; and no effusion of blood or perceptible injury at the parts on which the pressure was applied in strangling the animal. *Lungs*—collapsed, cepitating, and containing very little blood, excepting the lower portion of the left lobe, which bled freely on being incised. *Heart*—right and left auricles completely distended, as also the right ventricle, the left being nearly empty—coronary vessels beautifully injected, as also those of the pericardium. The diaphragm was greatly arched. *Abdominal Viscera*—mucous membrane of the stomach and intestines unusually vascular—spleen natural—liver of a deep red, and bleeding freely on being cut into—kidneys of a deep bluish color—bladder empty and contracted, its appearance being nearly natural—ascending and descending cavæ filled with black fluid blood—aorta nearly empty, and collapsed—mesenterie veins distended. *Brain*—vessels of the dura matter slightly injected—cerebral substance somewhat darker than usual—no blood effused into the ventricles, and none lost in opening the skull—spinal marrow perfectly natural.

*Experiment* 2.—In this experiment, which was likewise performed on a dog, the strangulation was effected in about nine minutes; and the evacuation of the urine and fœces followed immediately after the application of the hand. The examination was made twenty-four hours after death. *Neck*—veins highly distended—carotids empty, containing only a few drops of dark blood—mucous membrane of the fauces, larynx, trachea, and bronchia, deeply injected—tongue of a livid hue, and projecting slightly between the teeth—no froth either in the mouth or air passages. *Lungs*—collapsed, crepitous, and engorged with dark blood, the pulmonary veins being filled. *Heart*—Pericardium injected, and containing a large quanty of sanguinolent fluid—auricles filled with black blood, the venticles being nearly empty—coronary vessels distended—diaphragm arched, and highly vascular. *Abdominal Viscera*—engorged with blood, and the gastro-intestinal mucous membrane of a dark reddish tint—bladder empty, and contracted—ascending cava distended—aorta empty. *Brain*—dura and pia mater injected, the latter highly so—cerebral substance somewhat darker than naturally—a slight extravasation of blood at the base of the brain, accompanied by an engorgement of the vessels of that part. *Spinal marrow*—of a darkish color, and its membranes considerably injected.

*Experiment* 3.—Strangled a full grown rabbit by hanging with a cord, and allowed him to remain suspended about three quarters of an hour, although he expired in less than five minutes. *Head*—pericranium unnaturally vascular—dura mater perfectly transparent, and presenting not the slightest engorgement—pia mater partially injected—cerebral vessels natural—no effusion of blood into the brain or cranial cavity—eyes prominent—conjunctiva natural. *Mouth and Neck*—no froth in the mouth, or air passages, but the mucous membranes of these parts highly vascular—jugular veins filled with dark blood—carotid arteries empty. *Chest*—lungs collapsed, crepitous and slightly engorged, their color being a light red—cavities and vessels of the heart completely filled with blood—no serosity in pericardium. *Abdomen*—diaphragm arched—aorta empty—vena cava distended—liver of a deep red, and bleeding on being incised—spleen not engorged—mucous membrane of stomach and small intestines deeply injected, presenting a reddish aspect, approaching to vermilion—large intestines natural in their appearance—kidneys injected—bladder empty and contracted, its inner coating being of a light greyish tint.

*Experiment* 4.—In this experiment, in which the animal was strangled with the hand, the eyes were unusually prominent, and the tongue, which was of its natural color, projected between the teeth, there being no froth either at the mouth or nose—tbe large veins of the neck were distended, but the carotids were empty—the larynx was filled with frothy matter, and its lining membrane, throughout, was perfectly white. *Lungs*—these organs were but slightly congested, and when cut into gave vent to a frothy sanguinolent discharge: they did not cover the sides of the heart—the pulmonary arteries and veins were completely filled, as far as their point of entrance into the lungs—the four sides of the *heart* were occupied with black grumous blood, and the coronary vessels, as well as those of the pericardium, beautifully injected. *Abdominal Viscera*—the diaphragm was pushed towards the chest; and the abdominal organs, without exception, were perfectly natural in their appearance—the vena cava and mesenteric veins were distended; the gastro-intestinal mucous membrane was of its natural color; and the bladder empty, and contracted. *Brain*—this organ was of a much darker color than usual; the vessels of the pia mater were injected, but those of the dura mater perfectly empty. There was no water in the ventricles, nor any extravasation of blood—the spinal marrow had a healthy aspect, but the vessels of the pia mater were slightly injected.

The results of these and similar experiments, amounting in all to twelve in number, are almost precisely of the same import; yet, notwithstanding this, they unfortunately serve to show that they cannot be relied upon exclusively as proof of manual strangulation; and unless there be, at the same time, marks of violence of some kind or other upon the throat, the medical examiner would not be justifiable, I conceive, under any circumstances, to conclude that a murder of this sort had been committed; inasmuch as the general and more prominent signs of the different species of strangling are precisely alike. It does not appear to me, however, to be necessary that we should be able to make out the positive marks of fingers; on the contrary, it will be sufficient that there should be bruises or abrasions about the larynx or trachea. In the case of Mrs. Getter, it will be recollected, there was an indentation on the right side of the windpipe, resembling that made by a thumb or finger nail, and also an abrasion of the scarf-skin extending towards the left side of that organ; and it was only by taking these signs in connection with the bloated and suffused condition of the countenance, and the appearances revealed on dissection, that I felt myself authorized, on the trial, to give it as my decided conviction that the deceased had come to her end by manual strangulation; an opinion amply justified by the subsequent confession of the prisoner.

In none of my experiments was there any extravasated blood in the ventricles of the brain, or any extraordinary congestion of its vessels. In some, indeed, the cerebral substance was perfectly natural, as were also its enveloping membranes, the dura and pia mater. Hence, no reliance whatever, I conceive, should be placed upon any appearances that this organ may present in the case before us. Congestion of the brain, or extravasation of blood into its cavities, would much depend, in manual strangulation, on the manner in which the hand is applied upon the throat. If, for instance, the pressure is confined exclusively to the windpipe, it will be impossible for the brain to become engorged, because the blood in the jugular veins, being uninterrupted in its passage, will of course be able readily to find its way back to the heart; but if the return of this fluid is prevented by firmly constricting the vessels here referred to, apoplexy must, as a matter of course, be the result. In death from hanging this is probably almost always the case, from the manner in which the rope is fixed round the neck, whereas it would not necessarily follow in manual strangulation. In dissecting the body of John Harding, a soldier who committed suicide by hanging himself with a silk handkerchief, in March, 1833, I found the vessels of the dura and pia mater completely engorged, the cerebral substance of a very dark vascular aspect, and a large quantity of blood, amounting to no less than seven or eight ounces, extravasated into the ventricles, and on the base of the brain. He was a short, robust person, and the handkerchief was drawn so tightly round the neck as to prevent, very effectually, the return of the blood from the brain to the heart.

With respect to the distended state of the vessels of the neck, the engorgement of the lungs, and the loaded condition of the heart, they cannot be regarded as pathognomonic signs in the case before us, inasmuch as the same appearances are observed in death from hanging. I repeat it, therefore, that unless there are marks of violence about the throat, these symptoms should not be considered, under any circumstances, as positive indications of manual strangulation.

In a dissection made by Littre, a French physician, of a woman that had been strangled by the hand, there was a discharge of about an ounce of blood from the left ear, accompanied by a laceration of the tympanum; the vessels of the brain were unusually turgid; blood was extravasated into the ventricles, and also on the base of the cranium; the lungs were engorged, and their lining membrane unnaturally vascular. The cavities of the heart, however, were nearly empty, scarcely an ounce of blood being contained in the right ventricle, and this was *frothy*, like that of the lungs.* No mention is made, by the reporter of this case, as to whether there were any external lesions or not about the throat.

The marks of violence on the throat are sometimes very slight, yet, taken in connection with the appearances already referred to, they ought not to be considered as the less valuable in making up our opinion as to the probable cause of death. Metzger mentions the case of a young officer who was strangled in his bed by a soldier; yet the examining surgeon could find only one small spot on his windpipe, which the murderer afterwards confessed he had produced by violent pressure with his thumb.

Great discrepancy also not unfrequently occurs on the part of the witnesses, in relation to the number and extent of the marks about the throat, which the court and jury often find it extremely difficult to reconcile. Thus, in a man of the name of Beddingfield, who was murdered in England, in 1763, by manual strangulation, one of the surgeons testified that he saw the marks of a thumb and three fingers; the other, a thumb and four fingers; while a third witness, who also inspected the marks at the inquest, spoke of two only, "which looked as if the blood was set in the skin."† So also in the case of Mrs. Getter, one of the jury of inquest testified that the abrasion on the neck of the deceased was about the breadth of four fingers, another that it was three inches, and a third that it was only two inches. Little reliance should be placed upon such testimony; and as it is always the duty of the ex-

*Smith's Forensic Medicine, p. 244.

†Beck's Medical Jurisprudence, vol. ii. p. 49.

amining surgeon to note down every lesion about the body, however slight, no serious mistake will be likely to result even where the accounts are so extremely contradictory as they were in the cases to which I have just referred.

The time necessary to destroy life in manual strangulation does not, in all probability, exceed five or six minutes. In several of my experiments, I removed the hand from the throat at the end of three and a half and four minutes, and in every instance the animal speedily recovered, his first movements, however, being greatly confused, and like those of a person waking from a profound sleep. The safety of the animals in these cases was no doubt owing, at least in a great measure, to the facility with which the blood was enabled to return to the heart, thus preventing the brain from becoming apoplectic by an effusion of sanguinous fluid, or by over distention of its vessels. The ease with which a person may be killed in manual strangulation is strikingly exemplified in the death of Dr. Clench, which took place in London, in 1692. He was strangled in a hackney-coach by two men, whilst driving about the streets of the city, without any knowledge of the transaction on the part of the coachman, who afterwards found him kneeling down, with his head on the seat, quite dead, and a handkerchief bound round his neck, in which was a piece of coal, placed directly over the windpipe.* Getter was also but a short time in effecting the murder of his wife; for in the confession which he made on the day previous to his execution, he acknowledged that he was only a few minutes in accomplishing his diabolical purpose.

In concluding this paper, which has already been extended to an unusual length, it will not be amiss, perhaps, briefly to refer to some of those diseases, which, by suddenly inducing death, may give rise to appearances very similar to those characterizing manual strangulation. The most important of these are, apoplexy, hysteria, epilepsy, and intoxication.

In persons carried off by apoplexy the countenance is generally livid and suffused; the eyes red, and prominent; and

*Smith, op. cit. p. 239.

the internal organs, especially the brain, heart, and lungs, are engorged with black blood. These symptoms, considered in the abstract, might be very easily mistaken for those of manual strangulation; and the grounds for such an opinion would be still more plausible if the individual had died under circumstances calculated to excite suspicion. A man, for instance, may be suddenly overtaken with an apoplectic convulsion, in a place where there are hard or sharp objects, as various articles of furniture, if he be in a room, or stones or other bodies, if he be out of doors; and by falling upon these, he may not only receive considerable abrasions on the throat but even extensive bruises and wounds. In such cases the medical examiner would, of course, be extremely liable to err, and the only possible chance of arriving at any satisfactory conclusion, as to the probable cause of death, would be to enquire carefully into the habits of the deceased, his previous state of health, his predisposition to the disease in question, the spot where he has been found, and all the circumstances by which he was surrounded when discovered.

Hysteria usually comes on suddenly, and when the attack is violent, or the woman is predisposed to apoplexy, death may very easily overtake her out of doors or in some solitary place. The suffocating sensation which females generally experience in this disease, often induces them, as it were unconsciously, to grasp the throat, and hence, in attempts of this kind, it is not unreasonable to suppose that the marks of finger nails will sometimes be imprinted upon the skin of that region. The eyes of such as die in this manner are ordinarily prominent, and suffused; the face is livid, and swollen; the tongue protruded, or the jaws firmly locked; the fists are clenched; and the whole body is more or less contorted. The apoplectic form of this complaint—which is that here referred to—is commonly met with in robust, plethoric women, with short, thick necks, and strong and easily excited passions. These facts are of importance in a medico-legal point of view, and as such they ought never to be overlooked in our investigations.

With regard to death from epilepsy and intoxication, the symptoms would probably not be so strongly marked as in apoplexy and hysteria; yet as there is a *possibility* that they might simulate the appearances resulting from manual strangulation, it is but proper that the medical jurist should be on his guard, lest by a too hasty conclusion he may be instrumental in sacrificing the life of an innocent individual.

*Cincinnati, May,* 1835.

